# Patterns of Variation and Coordination in Shoot Traits Across Four Branching Orders of *Larix principis-rupprechtii*

**DOI:** 10.3390/life15060927

**Published:** 2025-06-09

**Authors:** Yang Yu, Huayong Zhang, Zhongyu Wang, Zhao Liu

**Affiliations:** 1 Research Center for Engineering Ecology and Nonlinear Science, North China Electric Power University, Beijing 102206, China; 120192132243@ncepu.edu.cn (Y.Y.); zhy_wang@ncepu.edu.cn (Z.W.); 2 Theoretical Ecology and Engineering Ecology Research Group, School of Life Sciences, Shandong University, Qingdao 250100, China; liuzhao9555@sdu.edu.cn

**Keywords:** intraspecific, shoot, branch orders, allocation, morphology, functional traits, coordination patterns

## Abstract

Intraspecific variation in functional traits can more accurately quantify plant responses to environmental changes and resource competition, while the plant economic spectrum provides a fundamental framework for understanding trait variation along environmental gradients. As the structural units of the aboveground branching system in woody plants, it remains unclear whether shoots exhibit a universal whole-plant economic spectrum and whether branch order significantly affects the patterns of trait variation and coordination. We collected 1551 shoots of *Larix principis-rupprechtii* to examine the patterns of trait variation and coordination from different branch orders to the whole-plant level. From the perspective of the plant economic spectrum, five functional traits were selected to represent the trade-off between structural and nutrient investment: the stem diameter (SD), stem length (SL), stem dry mass (SDM), specific stem length (SSL), and stem tissue density (STD). From different branch orders to the whole-plant level, allocation played a relatively more important role, and the patterns of pairwise trait correlations and trade-offs along the resource economic axis were consistent. Branch order did not strongly influence the correlations and degree of coordination within the shoot economic spectrum, as the whole-plant shoot economic spectrum was evident within each branch order. Our results support the hypothesis that the coordinated economic spectrum across branch orders forms an integrated whole-plant economic spectrum representing a “conservative–collaborative” resource management strategy. This strategy is robust to recent evolutionary changes (such as genotypic variation and even differences among shoots within the same species) as well as to variation across different branch orders.

## 1. Introduction

Functional traits are defined as morphological, physiological, and phenological characteristics that indirectly affect individual fitness by influencing three components of performance: growth, reproduction, and survival [1,2,3]. Uncovering the ecological processes underlying the assembly of natural communities is a central theme in plant community ecology, and plant functional traits serve as a vital link connecting the environment, individual plants, and the structure, processes, and functions of ecosystems [4,5]. As a result, functional traits have become a novel and widely applied approach and tool in related research.

Variation in functional traits results from the interaction between vegetation and the environment during the course of evolution, and it is central to understanding how plants respond to environmental gradients—one of the core issues in contemporary ecological research [6]. Plant functional trait variation objectively reflects a plant’s adaptability to external environmental conditions, and the values and combinations of these traits can quantitatively represent the significant influence of plants on ecosystem functional characteristics [7]. Generally, woody plants can adapt to environmental changes through three different types of adjustments: they can alter the relative allocation of biomass to roots, stems, and leaves; modify the morphological and structural characteristics of individual organs; or adjust the physiological properties within these tissues. Importantly, plants may adjust in all three aspects simultaneously [8]. Allocation can be quantified by the proportions of biomass invested in leaves (LMF, leaf mass fraction), stems (SMF, stem mass fraction), and roots (RMF, root mass fraction). Morphology can be expressed as the specific leaf area (SLA, total leaf area per unit leaf mass), specific stem length (SSL, stem length per unit stem mass), or specific root length (SRL, total root length per unit root mass) [8,9].

Functional trait variation can occur at multiple scales—within cells, leaves, individuals, populations, and species, between species, and even across environmental gradients and ecosystems [2,10]. Many previous studies on plant functional traits have typically used species as the unit of analysis. However, shoots, as the most dynamic structural units of the aboveground branching system in woody plants [11,12], perform multiple critical functions, including mechanical support, transport and storage, and reproduction [13,14,15]. Yet, studies specifically addressing trait variation in shoots are still scarce. Therefore, exploring the relative importance of adaptive adjustments at the levels of biomass allocation and morphology and determining how shoots respond to challenges under low or high nutrient availability would be a highly meaningful endeavor.

Intraspecific variation may originate from phenotypic plasticity or genetic diversity and fundamentally determines a species’ ability to adapt to environmental conditions [16]. Moreover, intraspecific variation in plant functional traits can influence, or even determine, life-history strategies and niche breadth, and it also affects species fitness, interspecific competition, community assembly, and ecosystem stability [10,17]. Incorporating intraspecific trait variation into ecological research allows for a deeper understanding of species interactions, population responses to spatiotemporal environmental gradients, and mechanisms of species coexistence and community assembly. It also enables a more accurate quantification of plant responses to environmental changes and resource competition [18,19]. *Larix principis-rupprechtii* is a key forest tree species in northern China and plays a crucial role in maintaining the ecological balance of local ecosystems [20]. Its growth condition and forest health directly impact soil conservation, hydrological cycles, and biodiversity [21]. At the same time, *L. principis-rupprechtii* is not only an important ecological resource but also has significant economic value [22]. Studying its growth patterns, wood properties, and the development and utilization of forest products is of great importance for promoting local economic development and sustainable use [23]. Therefore, determining the extent and patterns of intraspecific trait variation in the shoots of *L. principis-rupprechtii* is essential for understanding the key physiological and ecological processes of plants in different environmental contexts [24].

A set of interrelated functional traits forms an economic spectrum, representing the trade-off between adaptations that promote resource acquisition and those that promote resource conservation [25]. The most widely recognized spectrum is the leaf economic spectrum, which ranges from inexpensive, short-lived leaves (with a resource-acquisitive strategy, characterized by rapid returns on carbon and nutrient investment) to costly, long-lived leaves (with a resource-conservative strategy, characterized by slower returns on investment) [18,26]. The root economic spectrum (RES), on the other hand, defines trade-offs between water and nutrient transport versus mechanical support and defense against biotic attack and decomposition. Since the primary function of both shoots and fine roots is to acquire resources for growth, it remains unclear—when viewed through the lens of functional organ analogy—whether a branch economic spectrum exists as an aboveground counterpart to the RES and whether the relationships among shoot functional traits support the predictions made by the root economic spectrum.

Intraspecific variation in functional traits may deviate from the coordination among shoots due to changes in branch order, potentially weakening the overall tendency of the plant economic spectrum. Previous studies have shown that the patterns of vegetative growth and reproduction in shoots are related to the hierarchical position of terminal branches [11,27]. Many woody plants exhibit hierarchical shoot growth: with increasing branch order, the annual shoot length tends to decrease [28,29,30]. In other words, lower-order branches generally exhibit greater growth than higher-order branches across all varieties. Therefore, the patterns of trait covariation may depend on the classification of branch order. As revealed by a global analysis of 2031 species, leaf trait–trait covariation is not always consistent across taxonomic levels—such as species, genus, and family [31,32]. Similarly, the traits of shoots of different branch orders may have partially evolved independently. Moreover, few studies have explicitly tested trait coordination at the level of branch order. Thus, assessing whether branch order affects the intraspecific covariation in functional traits in shoots can offer new insights into understanding plant survival and adaptive strategies.

Previous studies have mostly focused on the relationship between the growth patterns of current-year shoots and factors such as light availability and structural position. For example, research has examined the effects of the branch structural position on the growth and branching patterns of *Cleyera japonica* shoots [28], the relationship between shoot growth patterns and light conditions or the structural position in young *Cleyera japonica* trees [30], and the crown development patterns in the *Fagaceae* family and structural variations in current-year shoots in response to light environments and tree height [33], as well as the mechanical and physiological ecological significance of morphology in young *Acer rufinerve* trees [34], including the vertical gradient of mechanical properties in branches. In addition, studies have addressed the allometric growth differences between current-year shoots and large branches in deciduous broad-leaved tree species [35], the variation in the branch structure and tree allometric growth with height in four deciduous tree species, and their relationship with maximum height [27]. More importantly, previous research has provided new insights into the molecular mechanisms of bud and shoot formation in *Larix olgensis* and laid a foundation for breeding new varieties [24]. However, these studies have not explored the relative importance of adaptation at the levels of biomass allocation and morphology, nor have they clarified how plants respond when challenged with low or high nutrient availability. Moreover, at a finer organ scale (such as tender branches), it remains unclear whether a universal whole-plant economics spectrum exists and whether the relationships among branch traits support the common predictions of the root economics spectrum (RES). Additionally, it is still unknown whether branch order strongly influences the coordination patterns of tender-branch traits.

Here, we measured five functional traits of terminal branches in the aboveground branching system of *Larix principis-rupprechtii*. The stem dry mass (SDM) reflects the investment of photosynthates in shoot growth [36]; the stem diameter (SD) is considered an important trait related to branch defense and lifespan extension [12]; and the stem length (SL) is regarded as critical for nutrient absorption and transport, especially under competition for aboveground resources [37]. The specific stem length (SSL) is considered the aboveground analog of the specific root length and serves as a core trait in aboveground plant economics, as it reflects the potential for leaf deployment (photosynthesis) per unit cost (biomass investment) [38]. The stem tissue density (STD) is a key functional trait in woody species, as it is associated with mechanical stability, hydraulic conductivity, and life-history strategies [39,40]. It has been regarded as an integrator of wood economics and a central axis of plant functional strategies [41]. This study aims to explore the patterns of variation and coordination in shoot functional traits across different branch orders. Specifically, we hypothesize the following: (i) in adapting to environmental changes, shoots generally exhibit greater flexibility, plasticity, and importance in adjusting biomass allocation rather than morphological traits; (ii) the coordination of functional traits across branch orders forms an integrated whole-plant economic spectrum that represents a “conservative–collaborative” resource management strategy, with trait coordination relationships consistent with and supportive of common predictions of the root economics spectrum (RES); and (iii) patterns of pairwise trait correlations and trade-offs along the resource economic axis differ between individual branch orders and the whole-plant level, with branch order strongly influencing trait coordination patterns.

## 2. Materials and Methods

### 2.1. Study Species

As a dominant tree species in cold-temperate coniferous forests, *Larix principis-rupprechtii* is mainly distributed in northern China [42]. *Larix principis-rupprechtii* is a strongly heliophilous tree species with a high dependence on sunlight. It grows well in the upper canopy or forest gaps. During the seedling stage, it can grow normally under strong light, while shading severely inhibits its growth and reduces its chlorophyll content. It has a clear apical dominance and a strong ability to compete for light resources. The species is relatively sensitive to water and is suitable for regions with annual precipitation of 600–1000 mm. It is highly cold-tolerant, capable of withstanding extremely low temperatures (below −30 °C). The optimal growth temperature is between 10 and 20 °C. It prefers deep, fertile, moist, and well-drained acidic loam or sandy loam soils. The suitable pH range is 4.5–6.5. It is intolerant of saline–alkaline soils and heavy clay. The tree is sensitive to phosphorus and nitrogen levels in the soil, and nutrient deficiency can restrict the growth of branches and leaves.

### 2.2. Study Area

**Figure 1:**
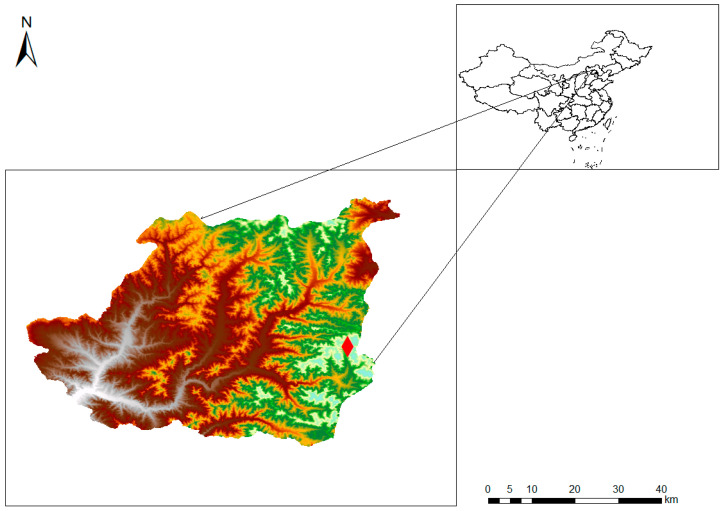
Location of the study area and sample site (the red diamond-shaped marker represents the sampling site).

This study was carried out in a plantation forest dominated by *Larix principis-rupprechtii* in the 2022 Winter Olympic core zone (40°57′ to 40°59′ N, 115°26′ to 115°27′ E), approx. 24 km northeast of the *Chongli* District, Zhangjiakou City, Hebei Province, North China. The age of the *Larix principis-rupprechtii* plantation is over 30 years. This study area is a typical mountainous area. The total forested area in the region is 1174 m^2^, with a forest coverage rate of 50.22%, making it one of the largest areas of natural secondary forest in Hebei Province. The study area falls under the East Asian continental monsoon climate. Winters are cold with low precipitation, frequent cold-air activity, and strong winds. Summers are warm, with rapid temperature increases and frequent heavy rainfall events. The average winter temperature is −12 °C, the average summer temperature is 18.4 °C, and the annual average temperature is 7.5 °C. The extreme maximum temperature reaches 42 °C, while the extreme minimum temperature drops to −34.7 °C. The maximum wind speed is 20 m/s. The area experiences early snowfall, thick snow accumulation, and a long snow retention period, with total winter snow accumulation reaching about 1 m. The average frost-free period is over 150 days. The multi-year average precipitation is around 426 mm, with a highly uneven temporal distribution. Most rainfall occurs from June to September, accounting for about 80% of the annual total, and it is often accompanied by localized rainstorms and hail. Interannual precipitation variation is significant, with alternating years of excessive and insufficient rainfall. The soil types in the study area include mountain brown soil, meadow brown soil, low-mountain cinnamon soil, sandy black soil, and herbaceous brown soil. Brown soils are mainly distributed on the shady slopes of mountainous forestlands, with a relatively high organic matter and nutrient content and a pH value of 6.5–7.0. The forest floor soil in mountainous areas generally occurs as a moderately thick layer and contains gravel and stones. Cinnamon soils are mainly found in relatively flat, rocky low mountains, located in the lower part of the brown soil zone. These soils occur in thin layers and have a lower organic matter content and a pH value of 6.5–7.5 (Figure 1).

### 2.3. Sample Collection

A 20 × 20 m plot was established within a larch plantation. In early August 2019, the height and diameter at breast height (DBH) of every tree within the plot were recorded. Healthy individuals were chosen from open areas within the plot that were not shaded by large trees, exhibited good growth, and were free from pests and diseases. Three randomly selected young trees had a diameter at breast height (DBH) of approximately 12.5 cm and a height ranging from 5.5 to 6.0 m. At the beginning of this study, branch systems from the upper crown of each tree were chosen to minimize differences in branch size. A total of 29 branch systems were selected (10, 10, and 9 systems from each tree).

**Figure 2:**
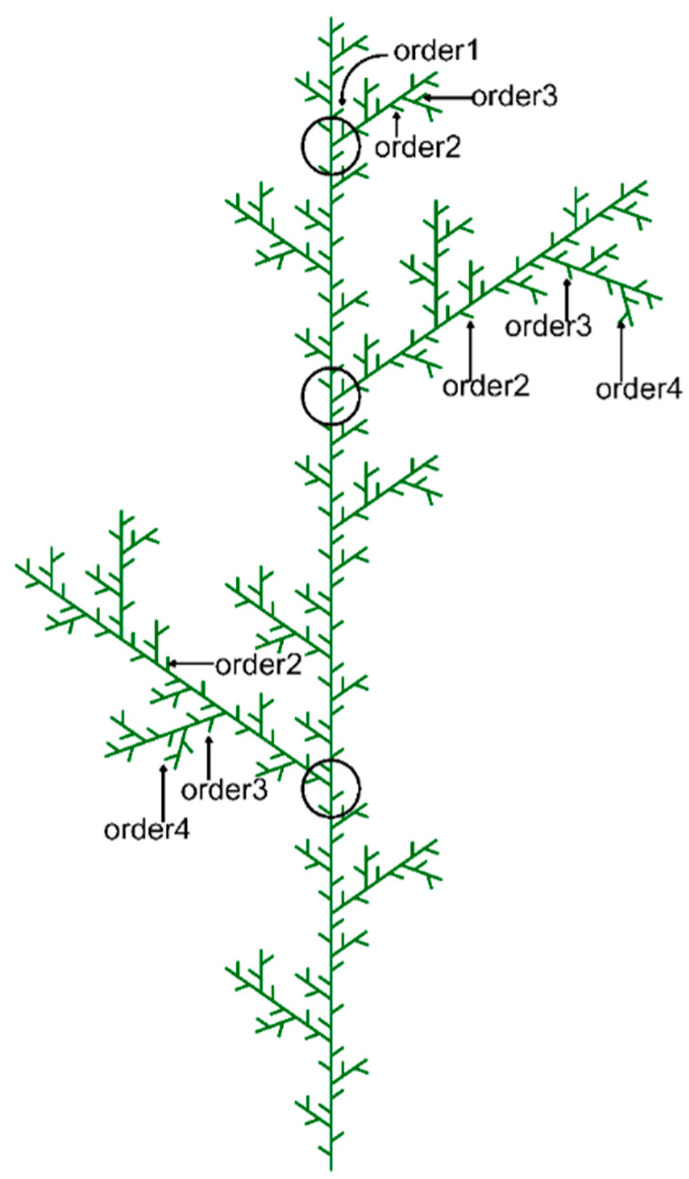
Diagram of the branching structure of *Larix principis-rupprechtii* and branch ordering in this study. The circles represent the whorls of the tree stem.

In this study, we focused on terminal branches, also referred to as current-year shoots. The terminal branch was defined as each terminal, unbranched segment of a branch (Figure 2), which is equivalent to the first-order branch in centripetal ordering systems, such as the Strahler system [43]. Two-dimensional diagrams were drawn to illustrate the branching structure of each branch system. The order numbers of branches and shoots were determined. The trunk was given order number 0, branches issued directly from the trunk were order 1, branches growing on order 1 branches were order 2, and so on (Figure 2).

### 2.4. Shoot Trait Measurements

We collected more than 20 intact shoots for morphometric measurements. Here, we mainly measured the most distal shoots, i.e., first-order shoots [44]. The diameter of first-order shoots was measured with a Vernier caliper. For shoot length, relatively short sections were measured using a Vernier caliper, and relatively long sections were measured using a tape measure. All thin slices and shoot segments were dried at 65 °C for 48 h and weighed (±0.01 g). The stem tissue density (STD) was estimated as the ratio of the shoot dry mass to its volume [8,9], assuming a cylindrical cross-section of the shoots. The specific stem length (SSL) was calculated by dividing the shoot length by its dry mass [8,9].

### 2.5. Data Analysis

For current-year shoots from different branch orders, we calculated the mean, median, minimum, maximum, and coefficient of variation (CV = standard deviation/mean × 100%) for each functional trait.

The Kruskal–Wallis test can absolutely be used for multiple groups with unequal sample sizes, which is one of its key advantages. It does not require each group to have the same number of samples, as the test is based on ranks rather than raw values, making it highly robust to unequal sample sizes (Table 1). One-way analysis of variance (ANOVA) was used to assess trait differences among branch orders.

Before conducting principal component analysis, we first used the KMO test and Bartlett’s test of sphericity to assess whether there was sufficient partial correlation among the variables in the sample data and whether it was appropriate to perform factor analysis or principal component analysis for dimensionality reduction. We have presented the KMO and Bartlett’s test results for the whole plant and for tender branches of different branch orders (Table 2). In addition, Pearson correlation analysis and principal component analysis (PCA) were conducted to quantify bivariate and multivariate trait relationships within each branch order. All statistical analyses were performed using R v.4.1.3 (R Core Team, 2022, New York, NY, USA). 

## 3. Results

### 3.1. Variations in Shoot Traits by Branching Orders

**Figure 3:**
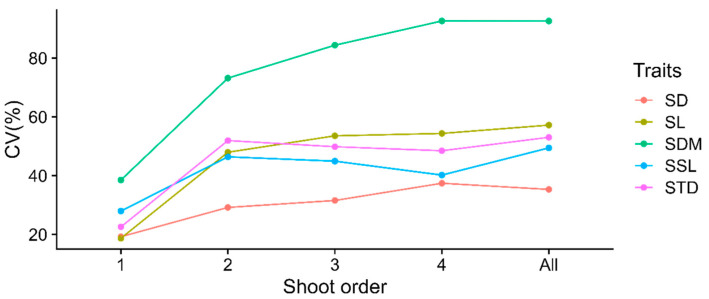
The average magnitude of shoot trait variability across four branching orders.

Combining all shoots across different branching orders, the magnitude of the coefficient of variation was from 35.34% to 92.63% in general. Specifically, the SDM showed the largest variation (CV = 92.63%), ranging from 0.01 to 1.49 g, but the SD had the smallest variation (CV = 35.34%), varying from 0.54 to 4.25 mm (Table 3). The coefficient of trait variation across most orders was ranked as SDM > SL > STD > SSL > SD, which was identical to the overall level. Moreover, the coefficient of variation of the five traits increased dramatically in the second order and then leveled off in higher orders to approximately the overall variation level (Figure 3).

**Figure 4:**
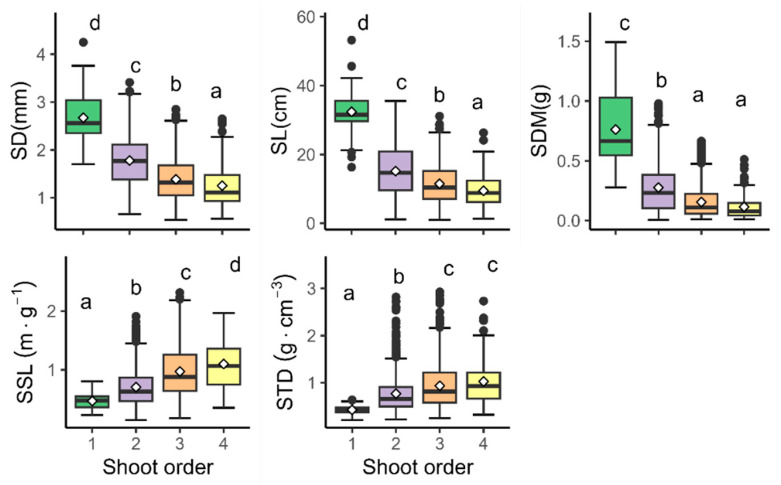
The distribution of shoot traits across different branching orders. Error bars represent ± SE of the mean. Significant differences are indicated by different lowercase letters at *p* < 0.05. Horizontal bars in each box represent the median of each shoot order, and diamonds correspond to the mean of the shoot order.

All five traits significantly differed by shoot orders (all *p*-values < 0.01) (Table 4). In addition, the SD, SL, and SSL differed significantly between each order. Meanwhile, the SDM and STD did not show significant differences between the third and fourth orders but did among the others. The tendency of variation differed in the relationship between the shoot traits and branch order. Specifically, the SD, SL, and SDM consistently decreased from the first to the fourth order and declined dramatically in the second-order shoots. In contrast, the SSL and STD increased gradually with increasing shoot orders (Figure 4).

### 3.2. Trait Correlations in the Shoot

**Figure 5:**
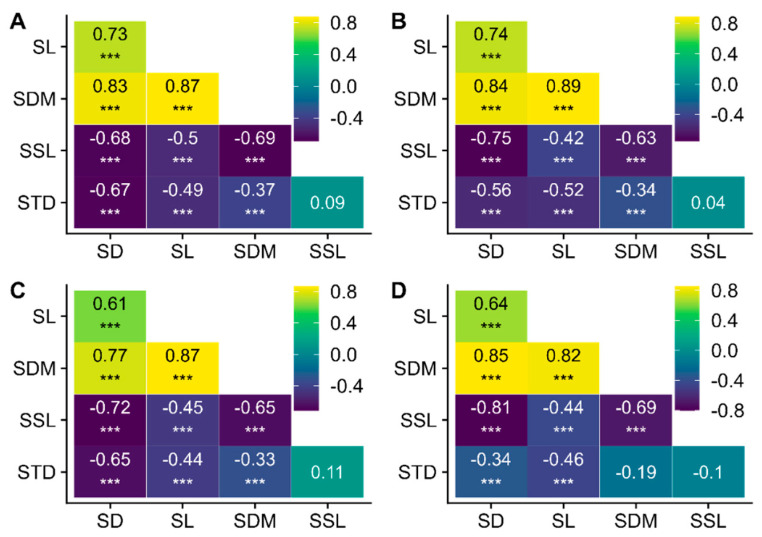
Correlations of shoot traits across different branching orders of *Larix principles-rupprechtii* (**A**–**D**). (**A**–**D**) correspond to order 1, order 2, order 3, and order 4, respectively. Correlations were significant at *** *p* < 0.001.

Here, we have generalized the patterns of shoot trait covariation across the four different branching orders in *Larix principles-rupprechtii*. When considered pairwise, almost all shoot traits were significantly correlated (Figure 5) among branching orders. Specifically, the three simple traits, including the SL, SD, and SDM, were positively correlated with each other (*p*-values < 0.001; Figure 5), and the three simple traits all showed negative correlations with the SSL and STD (*p*-values < 0.001; Figure 5). A nonsignificant relationship was found between the SSL and STD. The correlations among the three simple shoot traits were strengthened with increasing shoot order, and the relationship of other pairwise traits remained stable (Figure 5A–D).

### 3.3. Multivariate Coordination

**Figure 6:**
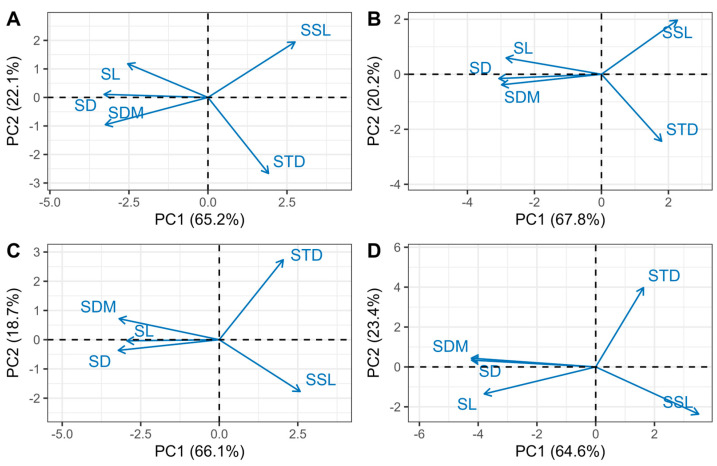
Coordination of shoot traits across different branching orders (**A**–**D**). (**A**–**D**) correspond to order 1, order 2, order 3, and order 4, respectively.

The cumulative variance contribution of PCA axes 1 and 2 for different branching orders exceeded 60%, effectively reflecting the relationships among bud functional traits (Figure 6). PC1 represents a structural axis, primarily determined by the SD, SL, and SDM, while PC2 is driven by the SSL and STD (Figure 6). The principal axes or components explained 67.3% of the total variation in the dataset (Table 5). The shoot diameter, length, and dry mass were negatively correlated with the primary variation axis. In contrast, other traits showed a positive correlation, both in the overall dataset and in the branching-order-specific subsets. The consistent directional loading of traits and the high percentage of variation explained by the principal axes within each branching order indicate a broadly universal coordination in shoot economic traits.

## 4. Discussion

### 4.1. The Relative Importance of Acclimation at the Level of Allocation and Morphology

The variation in plant functional traits is a response to continuous changes in the surrounding environment. Generally, woody plants can adapt to environmental conditions through adjustments at three different levels to maintain growth and reproductive functions: they can alter the relative allocation of biomass to roots, stems, and leaves; modify the morphology and structure of these organs; or adjust the physiological characteristics of these tissues. Importantly, plants may adjust all three aspects simultaneously [8]. Therefore, comparing the relative importance of these three levels of adjustment at the branch scale is a highly meaningful endeavor. In this study, allocation is quantified as the biomass invested in shoots, and morphology is represented by the SD and SL, SSL, and STD.

We report a small-scale estimation of the intraspecific trait variation (ITV) in the twigs of four branching orders of *Larix principis-rupprechtii* (Figure 2). Our results show that, both at the overall level and within each branching order, the coefficient of variation (CV) for the SDM is the highest among all functional traits. This indicates that, compared to modifying organ morphology and structure, plants exhibit greater flexibility and a stronger tendency to adjust their biomass allocation. In other words, allocation adjustments are more significant than morphological and structural adjustments. However, this finding contradicts previous studies, which generally suggest that plants have a greater capacity to adjust organ morphology than biomass allocation [9]. The discrepancy arises because prior studies focused on the whole-plant scale, whereas our study examines a much finer organ-scale perspective (e.g., shoots). Moreover, to maximize light capture and competitive advantage, terminal branch systems tend to allocate more resources to increasing twig numbers rather than modifying morphological traits [8]. Therefore, the high plasticity in biomass allocation within branch systems is a reasonable outcome. A significant challenge for future research would be to quantify the relative contribution of each adjustment level to the overall plant response and to understand how these trait adjustments are coordinated.

In the research results, we identified an interesting issue: regardless of whether at the overall level or the branch-order level, the SSL shows minimal response to changes in nutrient availability. This can be attributed to two factors. On the one hand, although low nutrient availability has a positive effect on the SSL, it simultaneously reduces the number of new shoots and the extension rate of the shoot length, which in turn negatively impacts the SSL (Figure 5). As a result, overall, there is little variation in the SSL within low-nutrient branch orders. Similarly, the variation in the SSL within high-nutrient branching orders follows the same pattern. Therefore, the effect of nutrient availability on the overall SSL is relatively mild [8]. On the other hand, as the terminal part of the aboveground branching system, the function of branches is not only to absorb nutrients and water but also to provide support and transportation. This requires a certain amount of support and transport tissue, which leads to the aboveground branching system of woody plants as a continuously expanding organ. Consequently, while maintaining transport capacity and supporting stability, shoots inevitably expand more rapidly in biomass than in length, exhibiting an allometric growth relationship between biomass and shoot length [26]. Since the SSL is the ratio of shoot length to biomass, as the branching system continues to expand, this ratio decreases. This explains why the response of the SSL to changes in the nutrient supply is so small. Therefore, in this study, it is important to describe the overall distribution of the SSL within the entire shoot system rather than just focusing on the average SSL of the branch system.

There are significant differences in traits among different branch orders, particularly between first-order shoots and the other three higher-order shoots, rather than among the three higher-order shoots themselves. This may be attributed to a hierarchical investment mechanism related to shoot growth and reproductive functions [29]. Specifically, lower-order shoots grow more but flower less, whereas higher-order shoots have limited growth but more vigorous flowering. In other words, plants prioritize shoot growth first and then allocate the remaining nutrient resources to reproductive functions, resulting in higher nutrient availability for lower-order shoots and lower nutrient availability for higher-order shoots. Lower-order shoots focus on vegetative functions, while higher-order shoots specialize in reproduction. This may also be a physiological explanation for apical dominance in woody plants [45]. Moreover, this is supported by our finding that the number of higher-order shoots is 21 times that of first-order shoots (Table 3). At the same time, this also explains why, for most traits, the coefficient of variation (CV) of second-order shoots increases sharply and then remains relatively stable in the subsequent higher-order shoots. Therefore, to better understand trait variation in shoots, it is essential to distinguish the different functional zones of shoots according to their branch order.

In this study, we found that the shoot diameter, length, and dry mass content exhibited a decreasing trend with increasing branching order. As observed by Arata Suzuki [28], in the upper canopy, the shoot length decreases with increasing branching order, whereas in the lower canopy, the differences in the shoot length among different branching orders are not significant. Additionally, Arata Suzuki also found that when the light environment is similar, lower-order branches tend to have a greater shoot length [28]. These similar findings can be attributed to three factors: besides the previously mentioned hierarchical investment pattern and apical dominance, an important additional factor is space availability [46]. The shoot length produced within a single growing season that does not overlap with other shoots is defined as the effective length (EL), which serves as an indicator of space availability. Previous studies have shown that both the effective length (EL) and space availability decrease with increasing branching order. Therefore, differences in space availability among branching orders may be a key factor contributing to the hierarchical growth of shoots.

At the same time, we found that the SSL and STD showed an increasing trend with increasing branching order. This may be due to the combined effects of apical dominance, hierarchical investment, and space availability, which cause higher-order shoots to experience environmental stress, such as reduced nutrient supply and increased shoot overlap. Since the core function of the SSL is resource acquisition, higher-order shoots tend to exhibit greater responses and variations under conditions of nutrient scarcity or environmental stress (e.g., reduced space availability). Similar to previous studies, roots generally respond to decreased nutrient availability by becoming thinner or by increasing the specific root length (SRL) and tissue density (RTD) [47]. Therefore, the positive correlation between the SSL, STD, and branching order is likely reasonable, as it helps maintain normal functional operation under environmental stress and intense competition.

### 4.2. Bivariate Relationships Among Shoot Traits

A close relationship between the SD, SL, and SDM was observed at the level of small-scale shoots (Figure 5), which is particularly useful for predicting how the SL and SDM of other shoot individuals change with the SD. The variation in the SSL results from two component traits: the SD and STD. The former determines the shoot length produced per unit shoot volume, while the latter determines the shoot volume produced per unit dry mass content. As described by Ostonen et al. [48], the SSL can be mathematically expressed using these two traits:

$$\mathrm{SSL}=4/(\pi \times D^{2}\times STD)$$

This equation implies that the SSL increases as *D* and/or *STD* decrease. The negative correlation between the SSL and *STD* can be explained mathematically since higher stem tissue density means greater stem mass per unit stem volume, which generally reduces the stem mass per unit stem length. The relationship between the SSL of these twigs and growth may suggest that if fine shoots with a high SSL ultimately acquire shorter nutrient uptake lengths or are less dependent on allocation, the differences in the SSL among woody plants are related to differences in growth strategies.

The negative correlation between the STD and simple traits, including the SD, SL, and SDM, suggests that the growth rate of first-order shoots decreases as the stem density increases, which may be due to two reasons. First, by definition, dense wood produces a smaller wood volume per unit biomass [35]. Second, dense wood may have a lower proportion of vessel elements, which could lead to reduced transpiration, photosynthesis, and biomass growth rates. Some studies have reported a significant negative correlation between stem density and species’ average diameter growth rates estimated from permanently marked individuals [49]. Given the numerous factors influencing growth and the different methods used to measure it, such results are not surprising. Comparisons across forest sites have found that slow diameter growth is associated with high stem density, such as in the Amazon rainforest [50] and Malaysia [51]. Notably, in some cases, high stem density may lead to higher annual growth rates.

### 4.3. Leading Dimensions of Shoot Trait Variation

We moved beyond the pairwise consideration of shoot traits to determine the extent to which twig economic traits covary in multidimensional trait space. This covariation can be quantified as the proportion of the total trait variation explained by the first principal axis in a principal component analysis. In a two-trait space, the principal axis is the long axis of the ellipse resulting from two correlated traits. In three-trait spaces, the principal axis is the long axis of an ellipsoid. In a multidimensional trait space, the principal axis describes the main axis of variation through a hyperellipsoid [26].

A remarkable 67.3% of all variation in the SD, SL, SDM, SSL, and STD across all shoots lays along the first principal axis in the five-trait space (Table 5). Because some of the residual 32.7% must be measurement variation, 67.3% represents a minimum estimate of the dominance of this single spectrum in explaining the variation across all shoots of *Larix principis-rupprechtii*. Multidimensional analyses, including five traits, similarly showed that the large majority of variation was explained with a single axis across four different shoot orders (Table 5). With the five traits included, more than 60% of all variation lays along the first principal axis across every shoot order (Table 5).

The contribution of each trait to the variation in the principal axis is represented by the loadings (or weights) assigned to each trait. The directionality of these loadings (Table 5) indicates that this principal axis can be regarded as a shoot economics spectrum. Similar to the root economics spectrum, there should also be a trade-off within shoots between rapid growth and fast resource acquisition versus longevity and conservative resource use [52,53,54]. Conservative shoots are expected to be thinner, have higher tissue density, and have lower nutrient concentrations, whereas acquisitive shoots should exhibit the opposite characteristics [54]. Shoots with a conservative trait syndrome are predicted to be more successful in harsh or resource-limited environments, while constructing acquisitive shoots may be more advantageous in resource-rich and highly competitive ecosystems [55]. Within branching order classes, the trait correlations along the primary axis of variation follow the same directionality as those in the overall dataset (Table 5). This consistency is particularly significant as it suggests that these key shoot traits exhibit uniformity across different branching orders. In most cases, the variation captured by the principal axes for different branching order groups is also similar to that for all shoots combined.

This study also found some relationships that were inconsistent with the predictions of the root economics spectrum (RES). According to RES predictions, the branch diameter and stem tissue density should be positively correlated. However, the study results showed a significant negative correlation between them, which contradicts the RES hypothesis. One possible reason for this contradiction is that the two anatomical traits of shoots—the stele tissue and cortex tissue [52,56]—exhibit an allometric growth relationship, with the cortex growing at a faster rate than the stele. As a result, as the radius increases, the proportion of the cortex becomes larger than that of the stele. Since the tissue density of the cortex is lower than that of the stele, the allometric growth relationship leads to an inverse relationship between the stem tissue density and diameter. Therefore, the observed relationship between the branch diameter and stem tissue density does not support the predictions of the root economics spectrum. Another possible explanation is that our study was limited to a relatively small scale or a restricted geographic range, which constrained the range of trait variation and may have masked broader trends [52,56]. Therefore, it is important for future research to use larger, global datasets to further examine these trait relationships.

## 5. Conclusions

In this study, we measured five functional traits of current-year shoots across different branching orders in *Larix principis-rupprechtii* to test the existence of a whole-plant economics spectrum. Our results indicate that, as part of the whole-plant resource economic strategy, traits across different branching orders evolved in the same manner. Furthermore, the branching order did not significantly affect the degree of trait coordination within the whole-plant economics framework. Therefore, we propose a successful plant adaptation strategy: even within a single species, there exists a trade-off between resource acquisition and conservation across different branching orders at the regional scale. Our key findings from this single species should encourage further studies on more tree species worldwide to test the generality of a strong economics spectrum in terminal branches.

## Figures and Tables

**Table 1:** The results of the Kruskal–Wallis test for five functional traits in shoots.

Traits	df	Χ^2^	*p*
SD	3	371.48	<0.001
SL	3	296.38	<0.001
SDM	3	350.74	<0.001
SSL	3	261.93	<0.001
STD	3	187.10	<0.001

**Table 2:** The results of the KMO and Bartlett’s tests for different branching orders.

Branch Orders	KMO Test	Bartlett’s Test		
		df	Χ^2^	*p*
Order 1	0.46	10	434.04	<0.001
Order 2	0.52	10	3186.681	<0.001
Order 3	0.49	10	2813.344	<0.001
Order 4	0.60	10	516.61	<0.001
Overall	0.54	10	7299.953	<0.001

**Table 3:** Summary of the five shoot traits across four branching orders of *Larix principles-rupprechtii*.

Traits	Order	*n*	Mean	SD	Median	Min	Max	CV%
	1	69	2.67	0.51	2.56	1.70	4.25	19.24
	2	713	1.78	0.52	1.77	0.66	3.41	29.20
SD (mm)	3	658	1.39	0.44	1.32	0.54	2.85	31.57
	4	111	1.25	0.47	1.11	0.56	2.65	37.43
	all	1551	1.61	0.57	1.54	0.54	4.25	35.34
	1	69	32.41	6.06	31.50	16.29	53.2	18.71
	2	713	15.15	7.27	14.66	1.10	35.5	47.98
SL (cm)	3	658	11.47	6.15	10.40	1.02	31.1	53.57
	4	111	9.42	5.12	8.80	1.30	26.3	54.38
	all	1551	13.95	7.98	12.49	1.02	53.2	57.21
	1	69	0.76	0.29	0.67	0.28	1.49	38.53
	2	713	0.28	0.2	0.23	0.01	0.98	73.20
SDM (g)	3	658	0.16	0.13	0.11	0.01	0.66	84.40
	4	111	0.12	0.11	0.08	0.01	0.51	92.64
	all	1551	0.24	0.22	0.16	0.01	1.49	92.63
	1	69	0.47	0.13	0.48	0.23	0.80	27.97
	2	713	0.71	0.33	0.63	0.15	1.91	46.42
SSL (m/g)	3	658	0.97	0.44	0.88	0.17	2.31	44.95
	4	111	1.10	0.44	1.06	0.35	1.97	40.21
	all	1551	0.84	0.41	0.74	0.15	2.31	49.46
	1	69	0.42	0.10	0.41	0.21	0.64	22.59
	2	713	0.77	0.40	0.65	0.22	2.82	51.95
STD (g/cm^3^)	3	658	0.94	0.47	0.81	0.25	2.93	49.86
	4	111	1.02	0.50	0.93	0.32	2.73	48.49
	all	1551	0.84	0.45	0.72	0.21	2.93	53.06

**Table 4:** Analysis of variance of five functional traits.

Traits	df	SS	MS	F	*p*
SD	3	144.36	48.121	206.75	<0.001
SL	3	30,851	10,283.8	234.51	<0.001
SDM	3	26.051	8.6837	281.95	<0.001
SSL	3	40.734	13.578	93.745	<0.001
STD	3	25.36	8.4533	46.053	<0.001

**Table 5:** Principal component analysis of the five functional traits.

	All	Order 1	Order 2	Order 3	Order 4
Variation explained (%)	67.3	65.2	67.8	66.1	64.6
Shoot traits	Loadings				
SD	−0.52	−0.53	−0.52	−0.51	−0.52
SL	−0.48	−0.41	−0.48	−0.47	−0.47
SDM	−0.50	−0.52	−0.51	−0.50	−0.52
SSL	0.39	0.44	0.38	0.41	0.43
STD	0.32	0.31	0.30	0.32	0.20

## Data Availability

The data that support the findings of this study are available from the corresponding author or the first author upon reasonable request.
